# Efficient Glare Suppression Network for Nighttime Images with Lightweight Parallel Attention and Ghost Convolution

**DOI:** 10.3390/s26123773

**Published:** 2026-06-12

**Authors:** Ruoyu Yang, Huaixin Chen, Sijie Luo, Zhixi Wang

**Affiliations:** 1 School of Resources and Environment, University of Electronic Science and Technology of China, Chengdu 611731, China; 2 Novel Product R & D Department, Truly Opto-Electronics Co., Ltd., Shanwei 516600, China

**Keywords:** nighttime images, glare suppression, lightweight network, ghost convolution

## Abstract

Aiming at the problems of glare interference, local overexposure and detail loss caused by artificial light sources such as vehicle lamps and street lamps in nighttime road scenes, as well as the challenges of existing glare suppression models with large parameters, high computational complexity and difficulty in deploying on edge devices, this paper proposes a lightweight glare suppression network (LGSNet) based on ghost depthwise separable convolution and Lightweight Parallel Attention. Based on the U-Net architecture, the network introduces ghost depthwise separable convolution blocks (GhostDSC) in the encoder and decoder, which generates ghost features through cheap linear transformations by exploiting feature map redundancy, significantly reducing model parameters and computational costs while maintaining feature representation ability. Meanwhile, a Lightweight Parallel Attention (LPA) module is designed in the decoder stage, which integrates channel attention and pixel attention in parallel, enhancing the network’s attention to glare regions and edge details with extremely low parameter increment to improve detail recovery accuracy. In addition, a joint loss function consisting of background loss, glare loss and reconstruction loss is constructed to collaboratively optimize glare suppression and detail preservation. Experimental results on the public Flare7K++ dataset and the self-built nighttime road glare dataset NRGD show that the proposed method has only 7.45 M parameters, much lower than standard U-Net and Uformer. It achieves competitive results on full-reference metrics such as PSNR, SSIM, LPIPS and no-reference metrics such as NIQE, BRISQUE, PIQE, and can effectively suppress various types of glare interference and restore obscured scene details. It achieves a superior trade-off between model complexity and enhancement performance, significantly reducing the parameter count and computational overhead compared to heavy baselines, thereby offering a highly efficient solution for resource-aware glare suppression tasks.

## 1. Introduction

Nighttime environments, characterized by insufficient illumination and complex light sources, are critical factors limiting the reliability of vision systems such as intelligent driving and security surveillance [1]. In images captured by practical devices including vehicle-mounted cameras, electronic rearview mirrors, and road monitors, intense point light sources such as headlights, street lamps, and billboards readily generate glare, halos, and overexposed regions [2], leading to local detail loss, texture blurring, and reduced contrast, which severely affect the accuracy of downstream tasks including object detection, lane recognition, and behavior analysis [3,4]. Particularly in the current era of rapid edge computing proliferation, lightweight, low-power, real-time image processing algorithms have become essential for engineering deployment. Therefore, research on nighttime glare removal methods that achieve both high suppression effectiveness and low computational cost holds significant theoretical value and practical relevance.

General low-light images suffer primarily from insufficient illumination, resulting in low brightness, poor contrast, and noise, but without strong light artifacts. Foggy images are degraded by atmospheric scattering, causing overall blur and reduced visibility with spatially smooth degradation. In contrast, nighttime road glare originates from high-dynamic-range artificial light sources such as headlights and street lamps, involving complex optical processes including internal lens reflections, surface scattering, and diffraction, which lead to localized overexposure, irregular halos, radial streaks, and occlusion of critical details. This fundamental difference in physical causes and degradation patterns necessitates dedicated glare-suppression techniques, which is the focus of this work.

In the field of low-light image enhancement and glare suppression, traditional methods primarily achieve brightness improvement and exposure correction based on theories such as histogram equalization [5], gamma correction [6], and Retinex decomposition [7]. While these methods are conceptually simple and computationally fast, they rely on handcrafted priors and exhibit poor adaptability to complex scenes, often suffering from color distortion, noise amplification, and incomplete glare suppression [8,9]. Deep learning has greatly advanced image restoration, where convolutional neural networks and Transformers play a key role due to their effective feature representation and global information modeling [10,11]. However, existing glare suppression networks tend to pursue higher restoration accuracy by continuously increasing network width and depth, resulting in a sharp increase in model parameters and slower inference speeds, making it difficult to meet the deployment requirements of resource-constrained platforms such as vehicle-mounted chips and embedded cameras [12,13].

In recent years, lightweight network design has become a crucial direction for the deployment of vision algorithms. Techniques such as depthwise separable convolution [14], ghost convolution [15], and channel pruning have been widely adopted to reduce model complexity. However, in glare suppression tasks, simply replacing convolutional layers leads to degraded detail recovery capability and inaccurate glare localization. Meanwhile, most lightweight methods focus solely on parameter compression, neglecting the characteristics of glare regions being irregular, having blurred boundaries, and being highly interwoven with normal regions [16], making it difficult for networks to accurately distinguish glare from background, ultimately resulting in insufficient suppression, excessive darkening, or detail blurring [17]. Therefore, constructing a network that is lightweight, efficient, capable of preserving details, and accurate in attention focus while maintaining glare suppression effectiveness remains an urgent challenge to be addressed.

To address the above challenges, the main contributions of this paper are as follows:

- Propose a lightweight glare suppression network LGSNet based on Ghost Depthwise Separable Convolution (GhostDSC) and Lightweight Parallel Attention (LPA), which achieves high-performance glare removal for nighttime road images while significantly reducing model complexity.
- Design the Ghost Depthwise Separable Convolution block (GhostDSC), which generates ghost features through cheap linear transformations by exploiting feature map redundancy, reducing the model parameters from 34.5 M of the standard U-Net to 7.45 M, significantly lowering computational overhead while preserving feature representation capability.
- Propose the Lightweight Parallel Attention (LPA) module, which fuses channel attention and pixel attention mechanisms in parallel, enhancing the network’s attention to glare regions and edge details with minimal parameter increment, effectively improving the local contrast and visibility of disturbed areas.
- To jointly optimize glare suppression and detail retention, we formulate a combined loss function comprising background loss, glare loss, and reconstruction loss. Comprehensive experiments on both the public Flare7K++ dataset and our self-built NRGD dataset confirm that the proposed method achieves superior performance in glare suppression, detail recovery, and lightweight design.

## 2. Related Work

### 2.1. Traditional Glare Suppression Methods

Glare suppression aims to eliminate or reduce light effects such as halos, flares, and radial streaks caused by intense light sources (e.g., headlights, street lamps, and reflections) in images, while restoring obscured scene details. It is an important research direction in the field of nighttime image enhancement. Unlike general low-light enhancement tasks, glare suppression requires addressing light effect elimination and detail reconstruction in locally overexposed regions, presenting higher technical challenges. Traditional methods have been extensively explored in this field.

To address flare spot artifacts caused by bright light sources in images, Vitoria et al. [18] developed an automatic detection and removal technique. It identifies flare regions using geometric, morphological, luminance, and color cues, while also considering lens optics and filter characteristics to refine candidate areas. An adaptive confidence strategy is introduced to accurately determine true flares and create corresponding masks, after which a sample-based inpainting approach restores the corrupted regions. This pipeline enables effective and fully automatic flare removal. In a related study, Zhang et al. [19] presented an image decomposition-based glare removal method to enhance both suppression quality and overall image clarity. Their technique separates an input glare image into a scene component and a glare component, compensates for brightness loss and color shifts, and then applies a local standard deviation-based contrast enhancement operation to recover fine details and improve contrast. This design effectively mitigates the typical color distortion and contrast degradation encountered under strong lighting conditions.

To tackle the quality degradation in coal mine surveillance footage caused by haze, poor lighting, and glare, Si et al. [20] developed a hybrid enhancement technique named SSR-BF, which integrates single-scale Retinex (SSR) with bilateral filtering (BF). The incorporation of bilateral filtering into the SSR framework helps suppress noise while preserving edge structures, leading to clearer surveillance images from coal mines. Meanwhile, Mandal et al. [21] introduced a region-based local enhancement approach. Their method splits each video frame into three separate regions and dynamically adjusts image patch sizes according to pixel brightness levels, using a block-based strategy tailored for low-light conditions. This design effectively prevents over-enhancement in both sky and dark areas, while emphasizing the brightness of roads and their surroundings. Additionally, a state judgment mechanism is incorporated to avoid unnecessary atmospheric light calculations, and local Gaussian filtering is applied along region borders, which reduces computational cost and boosts processing efficiency.

A dynamically tuned gamma correction strategy was presented by Rahman et al. [6], which improves image contrast and adapts well to diverse lighting scenarios. Chen et al. [22] developed a bi-histogram equalization method with minimum mean brightness error, achieving a favorable trade-off between contrast enhancement and detail preservation. Tang et al. [23], meanwhile, incorporated denoising and dehazing into the dark-channel prior framework, leading to noticeable suppression of halo artifacts.

Traditional glare suppression methods have achieved limited success in improving image quality. However, they are often constrained by several factors, including reliance on physical or rule-based models, poor adaptability to varying scenes, overall brightness reduction, halo artifacts, and high computational complexity. These limitations significantly hinder their application in complex and dynamic environments.

### 2.2. Deep Learning-Based Glare Suppression Methods

Deep learning has driven substantial progress in low-light image enhancement and glare removal in recent years [24,25,26]. For example, a lightweight Transformer-based enhancement model was proposed by Cui et al. [27]. Guo and Zhou et al. [28,29] used infrared-visible image fusion to improve nighttime visibility; and Huang et al. [30] addressed under- and overexposure by merging image inversion with exposure fusion. However, these methods are not specifically designed for glare suppression. A variational Retinex model for glow suppression was introduced by Liu et al. [31], and Sharma et al. [4] applied HDR to enhance dynamic range for glare mitigation. Jin et al. [3] developed a layer decomposition network with light-effect suppression, but its computational complexity remains high.

With the rapid development of edge computing, deploying deep learning models on resource-constrained edge devices has become an important research direction. In scenarios such as intelligent driving and security surveillance, edge devices including electronic rearview mirrors, dashcams, and smart cameras need to process complex visual information in real time under limited resources. Existing methods often pursue higher accuracy, resulting in large model sizes and high computational complexity, making it difficult to meet real-time requirements. For example, the Transformer-based Uformer model has 20.47M parameters, making it difficult to deploy directly on embedded devices [12].

Addressing the need for lightweight design, research on U-Net compression has been actively conducted. Zunair et al. [32] replaced standard convolutions with depthwise convolutions and introduce a sharpening filter to refine skip connections, achieving competitive performance in biomedical image segmentation with fewer parameters. Dong et al. [13] proposed a lightweight low-light denoising network using depthwise separable convolution, achieving an 88.34% reduction in parameters, an 8.88% reduction in runtime, and a 9.76% increase in frame rate on the BDD100K dataset. Liu et al. [33] proposed LIEDNet for low-light enhancement and deblurring. He et al. [34] proposed LEESDFormer with only 65K parameters and a processing time of just 8 milliseconds per image, fully demonstrating the potential of lightweight design on resource-constrained devices.

For glare removal tasks, Jiang et al. [35] proposed MFDNet, which decomposes light-polluted images using Laplacian pyramids to reduce computational cost, achieving superior performance on the Flare7K dataset [36]. These studies demonstrate that reasonable network architecture design can achieve model lightweighting without significantly sacrificing processing effectiveness.

Other researchers have explored glare suppression through different strategies. A combination of guided APSF simulation and gradient adaptive convolution was proposed by Jin et al. [16] for nighttime glare suppression. He et al. [37] introduced OENet to address overexposure, but its large size hinders embedded deployment. GR-GAN, which uses a glare attention detector, was developed by Niu et al. [17]; however, it requires paired supervision. Based on optical center symmetry prior, Dai et al. [38] presented a method for removing nighttime reflective flares. Wu et al. [2] combined physical modeling with semi-synthetic data to eliminate flares without real paired data, although generalization under extreme conditions remains an issue.

Low-light image enhancement and glare suppression have been substantially advanced by deep learning, which excels in detail recovery and scene adaptability. Nevertheless, current approaches still suffer from several drawbacks, including dependence on paired training data, poor generalization in extreme environments, and high computational demands. Particularly for nighttime road scenes with complex light sources, achieving efficient suppression without sacrificing detail while meeting real-time requirements remains a key challenge.

Existing lightweight methods for glare suppression still have shortcomings: simply replacing convolutional layers leads to degraded detail recovery and inaccurate glare localization; most methods neglect the irregular, blurred, and highly interwoven characteristics of glare regions [16], making it difficult to accurately distinguish glare from background, resulting in insufficient suppression, excessive darkening, or detail blurring [17].

In summary, although existing research has made significant progress, achieving a balance between restoration performance and computational efficiency remains challenging. Constructing a network that is lightweight, efficient, capable of preserving details, and accurate in attention focus while maintaining glare suppression effectiveness remains an urgent challenge.

## 3. Proposed Method

### 3.1. Overview

In nighttime road imaging, glare effects caused by artificial light sources such as headlights and street lamps represent one of the key factors degrading image quality. These glares typically appear in various forms, including halos, flare spots, and radial streaks, which not only cause local overexposure and texture blurring but also obscure critical information such as road edges and pedestrians, posing significant challenges to vision systems in intelligent driving and security surveillance. Although existing deep learning-based glare suppression methods have achieved considerable progress in restoration performance, their network structures have become increasingly complex, with model parameters often reaching tens of millions, making it difficult to meet the strict requirements of resource-constrained scenarios in terms of low power consumption and computational efficiency.

To mitigate the aforementioned issues, this work introduces a lightweight glare suppression network called LGSNet, designed from the perspectives of model compression and feature enhancement. In terms of architectural design, the network adopts an encoder–decoder framework and introduces ghost depthwise separable convolution (GhostDSC) blocks to replace traditional convolutional modules. By exploiting the inherent redundancy among feature maps, this module generates the remaining features from a small set of core features through cheap linear transformations, thereby compressing the total parameter count to 7.45M while preserving representational capacity—a reduction of approximately 78% compared to the standard U-Net. In the decoding stage, a Lightweight Parallel Attention (LPA) module is further designed, which combines channel-wise global selection and pixel-wise spatial focusing in parallel. This module enhances the network’s sensitivity to glare regions and edge structures with a negligible increase in parameters. Unlike generic lightweight networks designed for classification or detection (e.g., MobileNetV3, GhostNet), LGSNet is specifically optimized for glare removal by jointly addressing cross-channel color correlation, irregular boundary localization, and high-frequency detail recovery—challenges that are not explicitly tackled by existing lightweight restoration networks. It should be noted that glare artifact removal differs from general image restoration tasks in three aspects: cross-channel color correlation (e.g., reflective flares often appear purple or green), irregular and blurred boundaries, and the need for high-frequency detail recovery. Unlike MobileNetV3, whose depthwise separable convolutions decouple channel-wise and spatial operations and may weaken cross-channel interactions, our GhostDSC first generates a compact set of core features via a depthwise separable convolution (which already includes pointwise convolution for channel mixing) and then produces ghost features via cheap depthwise transformations. This preserves the cross-channel correlations that are crucial for identifying glare patterns. Furthermore, unlike advanced pruning techniques that passively reduce parameters without enhancing feature discriminability, our LPA module actively integrates channel attention (for spectral characteristics) and pixel attention (for spatial localization) in parallel. This active mechanism significantly improves sensitivity to glare regions and edge details.

Extensive experiments are conducted on the public Flare7K++ dataset and the self-constructed NRGD nighttime road glare dataset. Quantitative results show that LGSNet achieves competitive performance on full-reference metrics such as PSNR, SSIM, and LPIPS, as well as no-reference metrics including NIQE, BRISQUE, and PIQE, with particularly outstanding results on the perceptual metric PIQE. Visualization results further demonstrate that the network can adapt to glare interference of various colors and patterns and effectively recover occluded scene details. Ablation studies also validate the respective contributions of the GhostDSC and LPA modules to performance improvement. Overall, LGSNet significantly reduces computational overhead while maintaining high glare suppression quality, offering a feasible solution for nighttime image preprocessing toward edge deployment.

### 3.2. Paired Dataset Synthesis

The paired data synthesis method adopted in this paper follows the same physical model framework as previous works [12], primarily inspired by Wu et al. [2] and Flare7K++ [39]. The core concept of this method is to perform a weighted fusion of clean background images and flare patterns in the gamma-linear domain, thereby simulating the nonlinear response characteristics of real imaging processes. Specifically, the background image and flare pattern are first transformed from the gamma-encoded domain to the linear domain via gamma correction. A flare mask is employed to guide the blending process, where a mixing coefficient randomly drawn from a Gaussian distribution determines how the flare pattern is overlaid onto the background image. Finally, the synthesized image is converted back to the raw pixel domain through inverse gamma correction, and the pixel values are clipped to the valid range. Throughout the process, both the gamma value and the mixing coefficient are randomly sampled to ensure the diversity of the synthesized data. Following this procedure, we obtain paired training samples, each comprising a flare-degraded image and its corresponding clean background.

It should be noted that although the synthetic data generated by this method provide large-scale paired training samples, domain gaps still exist compared with real captured nighttime glare images. First, synthetic glare patterns are generated through idealized physical models, which may not fully reproduce the complex scattering and reflection effects in real lenses, resulting in overly regular textures. Second, real captured glare often exhibits higher dynamic range and more saturated colors due to sensor saturation and lens imperfections, while synthetic data tend to have more constrained intensity ranges. Third, real nighttime scenes contain multiple overlapping glare sources and complex background lighting conditions that are difficult to simulate accurately. These domain gaps may affect the generalization performance of models trained solely on synthetic data.

### 3.3. Framework of the Lightweight Glare Suppression Network

**Figure 1:**
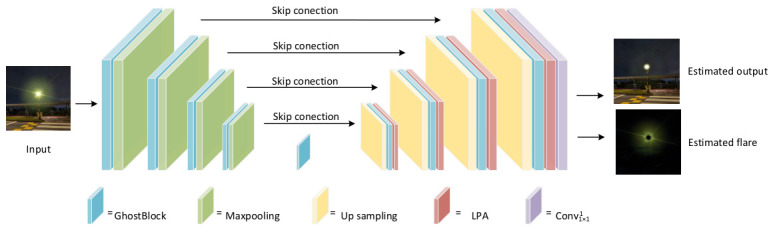
The structure of the proposed LGSNet.

As illustrated in Figure 1, the proposed lightweight glare suppression network consists of four core components: a GhostDSC-based encoder, a GhostDSC-based decoder, a Lightweight Parallel Attention (LPA) module, and an output head. Given an input image contaminated by glare, the encoder first performs multi-scale downsampling to progressively extract deep features at different scales. To effectively reduce the model parameter count, all standard convolutional blocks in both the encoder and decoder are replaced with GhostDSC modules. These modules exploit the redundancy inherent in feature maps to generate ghost features through cheap linear transformations, thereby significantly reducing computational overhead while preserving feature representation capability.

To enhance the network’s ability to perceive glare regions and recover occluded edge details, an LPA module is introduced after each layer of the decoder. This module adaptively strengthens the representation of key features by integrating channel attention and pixel attention mechanisms in parallel, enabling the network to more accurately focus on glare regions and their boundary structures. Subsequently, skip connections are employed to fuse the shallow spatial features from the corresponding encoder layers with the deep semantic features from the current decoder layer, forming a multi-scale feature pyramid that effectively mitigates the loss of detail during downsampling.

Finally, the output head maps the fused feature maps from the decoder into a 6-channel output. The first three channels produce the glare-free background image, while the remaining three channels generate the estimated glare mask, which explicitly separates the glare components. In addition, during network training, a joint loss function consisting of a background loss, a glare loss, and a reconstruction loss is introduced to collaboratively supervise the learning process from multiple perspectives, further enhancing the network’s comprehensive performance in glare suppression and detail preservation.

### 3.4. Ghost Depthwise Separable Convolution

**Figure 2:**
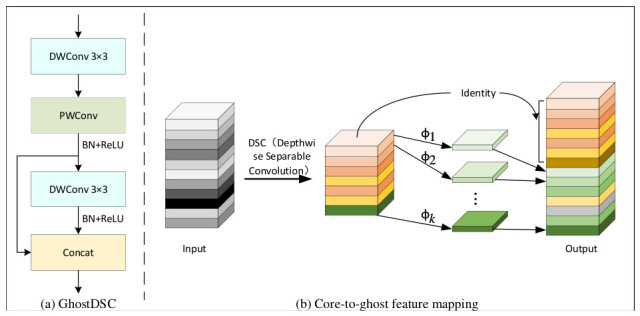
Illustration of the proposed GhostDSC module and core-to-ghost feature mapping. (**a**) GhostDSC module. (**b**) Core-to-ghost feature mapping. The input *X* is processed by a depthwise separable convolution to generate core features. Each core feature is then transformed by a cheap linear operation Φ (depthwise convolution) to produce multiple ghost features. The output is the concatenation of core features (identity) and all ghost features. This design reduces computational cost without sacrificing representational capacity.

Feature maps in deep neural networks often contain significant redundancy, with many feature maps being similar to each other, and can be generated from a few intrinsic features through simple linear transformations [40]. Inspired by GhostNet [15], this paper introduces this idea into depthwise separable convolution [14] and designs a Ghost Depthwise Separable Convolution (GhostDSC) block, whose structure is illustrated in Figure 2. The original GhostNet generates intrinsic features using a plain convolution (typically 1×1). In contrast, our GhostDSC employs a depthwise separable convolution (DWConv 3×3 + PWConv 1×1) for the same purpose. This modification further reduces parameters while enhancing cross-channel interaction through the pointwise convolution, which is crucial for capturing color shifts in reflective flares (e.g., purple or green artifacts). Standard depthwise separable convolutions (as in MobileNetV3) decouple channel and spatial operations but may weaken cross-channel correlation; GhostDSC explicitly retains channel mixing via the pointwise convolution step.

The core idea of this module is to first generate core features with a small amount of computation, then replicate more features through cheap transformations, and finally concatenate the outputs. When compared to standard convolution or traditional depthwise separable convolution, GhostDSC greatly lowers parameter quantity and computational cost. At the same time, it preserves a level of feature representation ability that is comparable to these methods. In recent years, mobile deep learning and lightweight model design have become hot topics in both academia and industry [41], and the design of this module follows this trend.

The detailed procedure is outlined below. Given an input feature map X, it first undergoes a 3×3 depthwise convolution to capture spatial information. Subsequently, batch normalization and a ReLU activation function are applied, yielding the intermediate feature map $F_{1}$:

(1) $$F_{1}=\mathrm{ReLU}\left(\mathrm{BN}\left({\mathrm{DWConv}}_{3\times 3}\left(X\right)\right)\right)$$

Next, a 1×1 pointwise convolution is performed on $F_{1}$ to adjust the channel dimensionality. This is followed by batch normalization and a ReLU activation, producing the core features $F_{2}$:

(2) $$F_{2}=\mathrm{ReLU}\left(\mathrm{BN}\left({\mathrm{PWConv}}_{1\times 1}\left(F_{1}\right)\right)\right)$$

This step generates only a portion of the total output features, thereby significantly reducing the computational cost. Assuming the total number of output channels is *C*, the core features account for only C/t (where t>1 is the compression ratio).

Then, for each core feature $f_{2}$, a cheap 3×3 depthwise convolution is performed to generate similar ghost features G:

(3) $$G=\mathrm{ReLU}(\mathrm{BN}\left({\mathrm{DWConv}}_{3\times 3}\left(F_{2}\right)\right))$$

Since these ghost features are derived from the core features, they do not require complex computation and can be obtained with only a simple depthwise convolution. Theoretically, the number of ghost features can be t−1 times that of the core features. Similar efficient module designs have also been applied to image restoration tasks, demonstrating the feasibility of reducing computational overhead while maintaining performance [42].

Finally, the core features $F_{2}$ and ghost features G are concatenated along the channel dimension to obtain the complete output feature map Y:

(4) $$Y=\mathrm{Concat}(F_{2},G)$$

The entire process does not change the spatial dimensions of the feature map but significantly reduces the parameter count and computational cost. Taking the convolutional blocks in standard U-Net as a reference, after replacing them with GhostDSC modules, the total parameter count of the encoder and decoder is reduced from 34.5 M to 7.45 M, a reduction of approximately 78%.

In this way, the GhostDSC module achieves model lightweighting while preserving feature representation capability. Subsequent experiments will verify the effectiveness of this module in glare removal tasks.

### 3.5. Lightweight Parallel Attention

**Figure 3:**
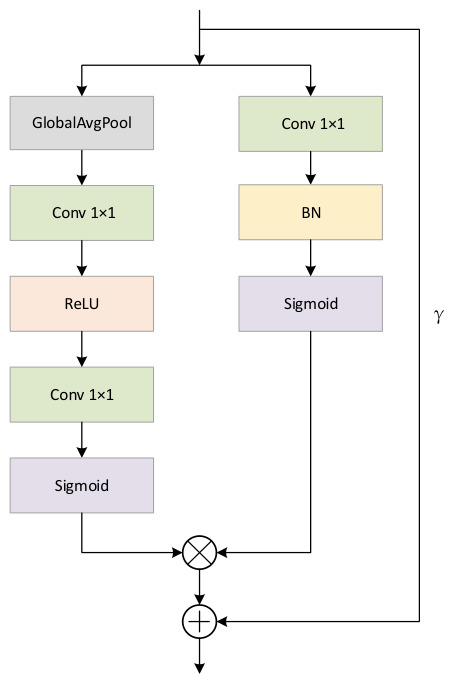
The structure of LPA.

On the basis of the lightweight U-Net, to further enhance the decoder’s attention to glare regions and fine details, this section introduces a Lightweight Parallel Attention (LPA) module. By integrating channel attention and pixel attention in parallel, this module effectively improves feature discriminability with almost no increase in computational cost, making it particularly suitable for recovering blurred boundaries and texture details caused by glare interference. Its structure is illustrated in Figure 3.

Studies in medical image segmentation have demonstrated that simultaneously modeling channel importance and spatial position responses can significantly improve segmentation accuracy [43]. Inspired by this finding, the LPA module is designed and introduced into the decoder stage to guide the network to focus more on feature channels and pixel regions that are critical for glare suppression. As shown in Figure 3, the LPA module adopts a parallel structure, computing channel attention maps and pixel attention maps separately, and then fusing them through element-wise multiplication. This design circumvents the potential information bottleneck inherent in serial architectures, while maintaining the independent representational power of each attention branch.

Channel attention is designed to capture global dependencies across different feature channels, emphasizing those most relevant to the current task. For an input feature map $X\in R^{C\times H\times W}$, global average pooling is first applied to aggregate spatial information, resulting in a channel descriptor $g\in R^{C\times 1\times 1}$. This descriptor is then passed into a lightweight sub-network that comprises two 1×1 convolutional layers with a ReLU activation placed between them, enabling the modeling of nonlinear inter-channel relationships. Finally, a Sigmoid function is applied to generate the channel attention weights a. This process is defined as follows:

(5) g=GlobalAvgPool(X)

(6) $$a=\sigma ({\mathrm{Conv}}_{1\times 1}\left(\delta \left({\mathrm{Conv}}_{1\times 1}\left(g\right)\right)\right))$$

where δ(·) refers to the ReLU activation function, σ(·) denotes the Sigmoid function, and ${\mathrm{Conv}}_{1\times 1}$ represents a convolution with a kernel size of 1×1. Subsequently, the input feature map is element-wise multiplied by the channel attention weights to produce the channel-enhanced features $X_{\mathrm{ca}}$:

(7) $$X_{\mathrm{ca}}=a⊗X$$

where ⊗ denotes element-wise multiplication.

Pixel attention adaptively adjusts the importance of each spatial position, enabling the network to focus on glare regions and their boundaries. This branch also employs two 1×1 convolutional layers, with the first followed by a ReLU activation and the second followed by a Sigmoid function, directly generating the pixel attention map $P\in R^{1\times H\times W}$:

(8) $$P=\sigma ({\mathrm{Conv}}_{1\times 1}\left(\delta \left({\mathrm{Conv}}_{1\times 1}\left(X\right)\right)\right))$$

The input feature map is then multiplied element-wise with the pixel attention map to obtain the spatially enhanced features $X_{\mathrm{pa}}$:

(9) $$X_{\mathrm{pa}}=P⊗X$$

The LPA module combines the outputs of the two attention branches via element-wise multiplication, while a learnable residual connection is introduced to enhance training stability. The resulting output $X_{\mathrm{out}}$ is formulated as

(10) $$X_{\mathrm{out}}=X_{\mathrm{ca}}⊗X_{\mathrm{pa}}+\gamma \cdot X$$

where γ is a learnable scalar parameter initialized to 0, allowing the module to initially behave as an identity mapping and gradually enhance the attention effect during training. This parallel fusion mechanism simultaneously leverages global statistical information across channels and local context at the pixel level, effectively improving feature sensitivity to glare regions. The residual connection with a learnable coefficient γ (initialized to 0) is essential for training stability. It mitigates gradient vanishing caused by nonlinear activations and multiplications in the attention branches, and preserves the original input information, ensuring that the network maintains global structure even when attention weights become extremely small in some regions.

The design of the LPA module is centered on lightweighting. The channel attention branch contains only two 1×1 convolutions, and the pixel attention branch does the same, resulting in a very limited increase in parameters. For comparison with serial channel-spatial attention mechanisms (e.g., CBAM), typical serial approaches apply channel and spatial attention sequentially (channel → spatial or spatial → channel). This may create an information bottleneck, where the second branch operates on features already gated by the first branch. In contrast, our LPA adopts a parallel design: channel attention and pixel attention are computed independently from the same input and then fused via element-wise multiplication. This preserves the full representational capacity of both branches and avoids sequential dependency. Furthermore, a learnable residual coefficient γ (initialized to 0) is introduced to stabilize training, which is not commonly found in existing channel-spatial attentions such as CBAM.

The feature redundancy captured by GhostDSC supplies the LPA module with a rich set of candidate features that contain high-frequency edge information. LPA then applies channel and pixel attention in parallel to selectively enhance the most relevant components, thereby improving the localization and restoration of high-frequency glare edges. To maximize the benefits of this synergy, the LPA module is placed after the GhostDSC blocks at four stages of the decoder, corresponding to resolutions of H/8, H/4, H/2, and *H*. This multi-scale placement ensures that features at each decoder layer undergo attention-based refinement before being fed into subsequent upsampling or the final classification layer, progressively recovering the details lost during downsampling. This design is particularly beneficial for improving the reconstruction quality of glare edges, enabling the network to recover fine details even under heavy glare occlusion.

### 3.6. Loss Function

To jointly optimize glare suppression and detail preservation, we employ a composite loss function that integrates three components: a background loss $L_{B}$, a glare loss $L_{F}$, and a reconstruction loss $L_{rec}$. The overall loss is computed as

(11) $$L=w_{1}L_{B}+w_{2}L_{F}+w_{3}L_{rec}$$

where weighting factors $w_{1}$, $w_{2}$, and $w_{3}$ are empirically set to 0.5, 0.5, and 1.0, respectively.

Let Φ denote the proposed glare suppression network. Given an input image I corrupted by glare, Φ produces two outputs, the estimated clean background ${\hat{I}}_{0}$ and the estimated glare layer $\hat{F}$:

(12) $$\left({\hat{I}}_{0},\hat{F}\right)=\Phi \left(I\right)$$

The background loss encourages the recovered background to be close to the ground truth, while the glare loss does the same for the estimated glare component. Both are defined as a combination of L1 distance and VGG perceptual similarity:

(13) $$L_{B}=L_{1}\left({\hat{I}}_{0},I_{0}\right)+L_{\mathrm{vgg}}\left({\hat{I}}_{0},I_{0}\right),\;L_{F}=L_{1}\left(\hat{F},F\right)+L_{\mathrm{vgg}}\left(\hat{F},F\right)$$

where $I_{0}$ and F represent the ground-truth background and glare images, respectively.

The reconstruction loss enforces the consistency between the input and the sum of the two estimated components, which is essential for physically meaningful decomposition:

(14) $$L_{rec}={∥I-\mathrm{Clip}\left({\hat{I}}_{0}⊕\hat{F}\right)∥}_{1}$$

where ⊕ denotes element-wise addition in the gamma-decoded domain. Specifically, the images are first linearized via gamma decoding using a randomly sampled gamma value γ (sampled from the range [1.8,2.2]). The addition operation is then carried out, and finally the Clip(·) function is applied to clip the resulting values to the [0,1] range to ensure valid pixel intensities. ${∥\cdot ∥}_{1}$ represents the L1 norm.

## 4. Experiments

### 4.1. Experimental Setup Details

#### 4.1.1. Model Training Details

Our model was trained using two NVIDIA GeForce RTX 3090 GPUs (NVIDIA Co., California, USA) (CUDA 11.2, driver 460.32.03). The Adam optimizer was employed with parameters $\beta_{1}=0.9$ and $\beta_{2}=0.99$. A batch size of 2 was used, and each input image was resized to 520×520 pixels. The training started with a learning rate of $10^{-4}$, which was reduced by half after 200,000 iterations. A total of 300,000 iterations were performed. To ensure training stability and convergence, an exponential moving average with a decay factor of 0.9 was applied.

#### 4.1.2. Datasets

**Figure 4:**
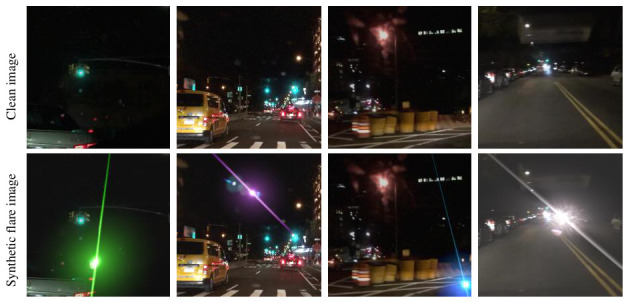
Examples of paired samples in the NRGD dataset.

The publicly available Flare7K++ dataset [38] is utilized for both training and evaluation. Moreover, to assess the generalizability of the proposed glare suppression model, we conduct cross-dataset validation on the NRGD dataset [12], which serves strictly as an unseen test set (i.e., no training or fine-tuning is performed on NRGD). NRGD is specifically designed for real-world nighttime driving scenarios, capturing videos using a vivo S19 smartphone (manufactured by Vivo Mobile Communication Co., Ltd., Dongguan, China) equipped with a 50-MP OIS-stabilized main camera and a 0.6× ultra-wide setting to cover strong light sources (e.g., headlights, streetlamps). Videos were recorded at 1920 × 1080 resolution across diverse urban environments. Frames were extracted at 3 s intervals, yielding approximately 500 real-world images. These images encompass complex glare patterns, low-light conditions, and dynamic traffic elements, posing a rigorous challenge for generalization. Representative examples of these scenarios are shown in Figure 4. Comprehensive details regarding acquisition protocols and dataset characteristics can be found in [12].

#### 4.1.3. Evaluation Metrics

To provide a thorough evaluation of our method, we employed four full-reference and three no-reference metrics, each capturing different aspects of image quality.

The full-reference metrics compare the restored images against ground-truth references. These include Mean Squared Error (MSE), Peak Signal-to-Noise Ratio (PSNR) [44], Structural Similarity Index (SSIM) [45], and Learned Perceptual Image Patch Similarity (LPIPS) [46]. PSNR measures pixel-level fidelity, with higher values reflecting lower distortion. SSIM evaluates luminance, contrast, and structural consistency, where values approaching 1 indicate better structural preservation. LPIPS computes feature-space distances using a pre-trained deep network, where lower scores indicate higher perceptual similarity to human vision.

The no-reference metrics operate solely on the enhanced images without requiring ground-truth data. We use the Natural Image Quality Evaluator (NIQE) [47], the Blind/Referenceless Image Spatial Quality Evaluator (BRISQUE) [48], and the Perception-based Image Quality Evaluator (PIQE) [49]. NIQE measures how closely an image matches the statistical properties of natural scenes, making it sensitive to noise and color shifts. BRISQUE evaluates blur and noise based on locally normalized luminance statistics. PIQE mimics human visual perception and is particularly effective at detecting local distortions such as halos and artifacts, which are common in nighttime glare suppression tasks.

### 4.2. Quantitative Comparison with State-of-the-Art Methods

The effectiveness of LGSNet is demonstrated through comparison with three deep learning models: EnlightenGAN [50], U-Net [39], and Uformer [39], as well as three traditional methods, namely Adaptive Histogram Equalization (CLAHE), Adaptive Gamma Correction (GAC), and Multi-Scale Retinex with Color Restoration (MSRCR). The choice of these baseline methods is justified as follows. U-Net and Uformer are adopted following the most representative work in the glare suppression field, Flare7K++ [39], where they serve as primary baselines for glare removal. EnlightenGAN is a classic unsupervised method for low-light enhancement and has been widely used for nighttime image enhancement. As for traditional methods, CLAHE, GAC, and MSRCR are representative approaches for contrast adjustment, gamma correction, and Retinex-based enhancement, respectively, providing a comprehensive comparison across different categories of image enhancement techniques.

As shown in Table 1 and Table 2, the proposed method achieves excellent quantitative results on both the Flare7K++ and NRGD datasets. Regarding perceptual quality, our method achieves the best PIQE scores across both datasets (36.5721 on Flare7K++ and 35.8103 on NRGD), and also obtains competitive results on the NIQE metric. This suggests that the enhanced images align more closely with human visual perception and possess greater naturalness. For traditional metrics like PSNR and SSIM, our method ranks second or third, falling only slightly behind the top-performing Uformer (by less than 1.5 dB in PSNR and within 0.02 in SSIM), while clearly surpassing other baselines such as U-Net. It is noteworthy that on the NRGD dataset, our method exhibits a trade-off across different perceptual metrics. While larger models like Uformer achieve superior performance on the deep-feature-based LPIPS metric (0.1626 vs. 0.4466), our method attains the best scores on the no-reference metrics PIQE and NIQE. This suggests that although our lightweight design may limit the capacity to perfectly reconstruct deep feature similarities, it effectively enhances local naturalness and reduces artifacts, which are critical for practical nighttime driving scenarios. Within the constraints of a highly efficient architecture, our method achieves a balanced performance, excelling in metrics related to local distortion and naturalness.

Furthermore, Table 3 presents a comparison of processing speed and model parameters among different methods. Although traditional image processing methods such as CLAHE and GAC have advantages in speed (20.49 fps and 25.62 fps, respectively), they lack deep understanding of image content and struggle to effectively handle complex glare scenes. Among deep learning methods, the proposed method achieves a processing speed of 2.20 fps, which is superior to U-Net (1.42 fps) and Uformer (1.55 fps). Moreover, its parameter count is only 7.45M, which is much lower than that of U-Net (34.51M) and Uformer (20.47M), and also better than EnlightenGAN (8.636M). That said, 2.20 fps does not satisfy the strict real-time requirement expected in live autonomous-driving or active ADAS pipelines. In practice, however, glare removal is positioned here as an offline or asynchronous preprocessing stage (e.g., frame-downsampled capture, key-frame enhancement, or post-event analysis), where a 2.20 fps throughput is still acceptable because it can operate on selected frames without blocking the primary detection/tracking branch running at higher rates. In summary, the proposed method achieves the best or near-best results on perceptual quality metrics with the smallest parameter count and acceptable processing speed, fully demonstrating its favorable balance between model lightweighting and enhancement effectiveness, and validating its effectiveness and practicality in glare removal tasks.

### 4.3. Qualitative Comparison with State-of-the-Art Methods

**Figure 5:**
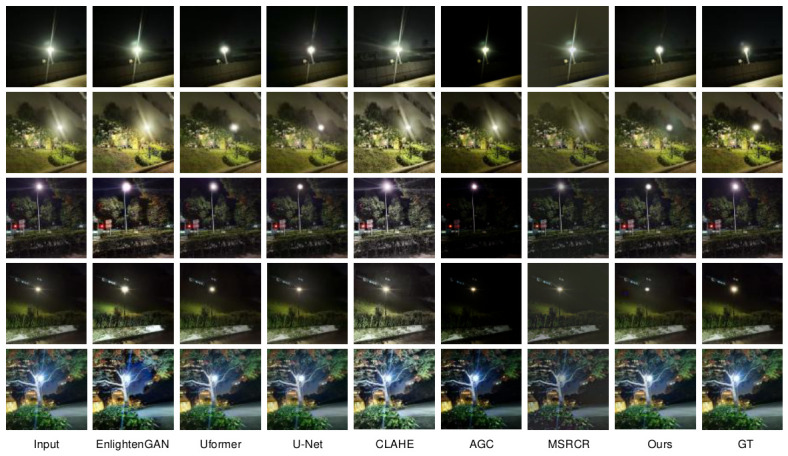
Comparison of visual results of different methods on the Flare7K++ dataset.

Figure 5 presents the visual comparison of different methods on the Flare7K++ dataset. EnlightenGAN and Adaptive Histogram Equalization (CLAHE) only achieve overall brightness enhancement and even diffuse the glare regions. Adaptive Gamma Correction (AGC) exhibits a certain degree of glare suppression but fails to completely eliminate large radial glare. The MSRCR method leads to overall image blurring, resulting in a gray mask-like effect. The U-Net method can suppress glare to some extent, but residual radial streaks remain in the white radial glare region in the first image. The Uformer method achieves performance comparable to our proposed method, both demonstrating favorable glare suppression effects. However, as can be seen from the suppression results of the white street lamps in the second, third, and fourth images, although Uformer shows superior suppression compared to other methods, radial halos around the light sources still remain. In contrast, our method suppresses the glare to a very small region around the source, making the surrounding details much clearer. This aligns well with our competitive PIQE score, which penalizes halos and local overexposure.

**Figure 6:**
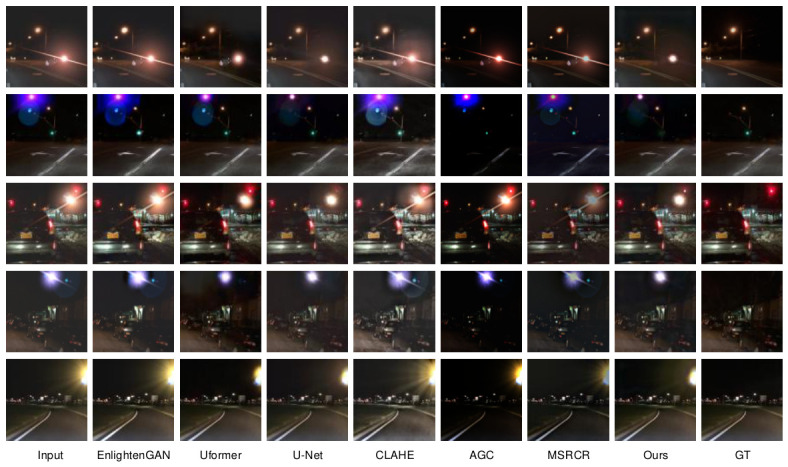
Comparison of visual results of different methods on the NRGD dataset.

Figure 6 shows the visual comparison of different methods on the NRGD dataset. EnlightenGAN and CLAHE still only achieve overall brightness enhancement, while AGC and MSRCR suppress glare by globally darkening the images, albeit with weak effectiveness. Uformer and U-Net achieve comparable performance to our method, but Uformer fails to effectively suppress the blue light artifact beneath the purple glare in the second image, whereas both U-Net and our method achieve satisfactory suppression in this region.

**Figure 7:**
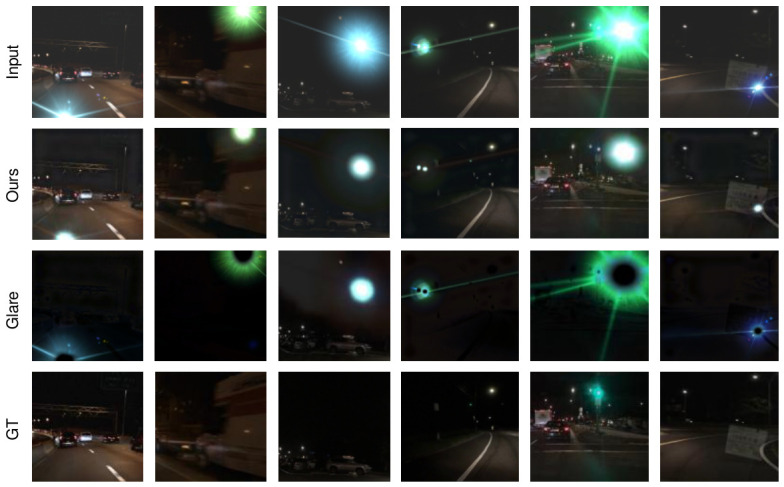
Visualization results of the proposed method on the NRGD dataset.

Figure 7 presents the visual results of our method on the NRGD dataset. For various colors of glare in nighttime road scenes, such as the green glare in the second image and the blue glare in the third image, our method effectively suppresses the radial and scattering glare around the light sources and accurately predicts the location, shape, size, and color of the glare. These findings clearly validate the efficacy of our approach for nighttime glare suppression.

**Figure 8:**
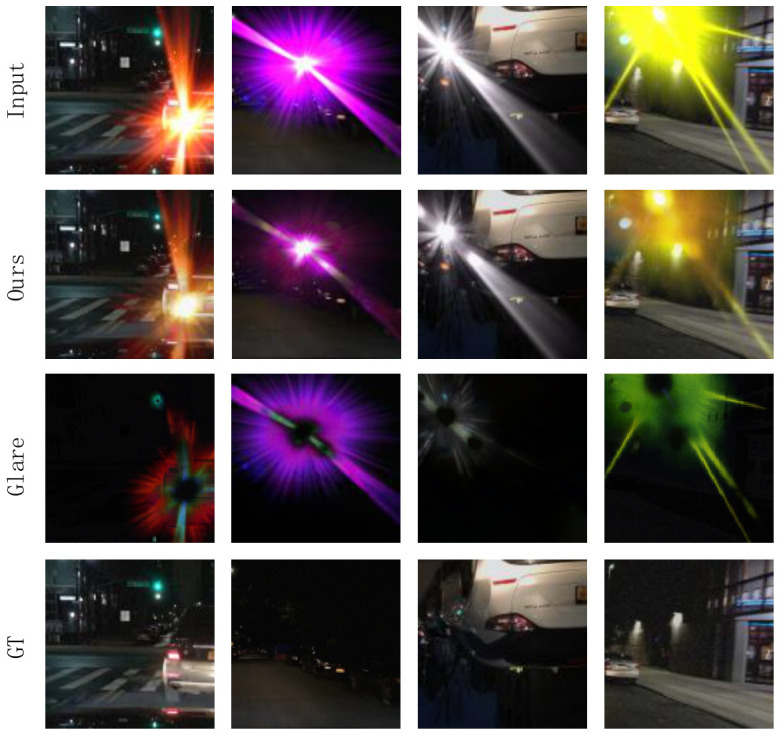
Visualization results of the proposed method on extreme scenarios of the NRGD dataset.

To further explore the limitations of our method, we additionally tested it on a subset of the NRGD dataset containing extreme strong glare cases that cause severe structural blurring. As shown in Figure 8, under such challenging conditions, the glare suppression effect becomes notably weaker: residual halos remain visible, and dark-area details (e.g., road markings, car outlines) are only partially recovered. We attribute this performance drop to two main factors. First, the training data (Flare7K++) is dominated by synthetic glare patterns generated from idealized physical models, which do not sufficiently cover the complexity of real-world extreme multi-source overlapping glare. Second, the lightweight design (GhostDSC + LPA) inevitably trades off some modeling capacity for high parameter efficiency. This analysis helps delineate the applicability of our method (common nighttime glare) and its limitations (degraded performance under extremely complex glare), while pointing to future work such as collecting more extreme-case data and exploring more powerful models.

### 4.4. Ablation Study

To evaluate the contribution of each component, we perform an ablation study on the NRGD dataset, with the results summarized in Table 4. As shown in the table, removing the GhostDSC module (replacing it with standard convolution) results in a slight improvement in pixel-level fidelity (PSNR: 22.1337 vs. 21.9224) and deep feature similarity (LPIPS: 0.4456 vs. 0.4466). This is expected because standard convolutions possess more learnable parameters, allowing for a tighter fit to the training distribution. However, this comes at the cost of significantly increased computational complexity and a notable degradation in image naturalness. Specifically, the full model with GhostDSC achieves substantially better performance on no-reference metrics, reducing NIQE from 7.8616 to 7.1397 and PIQE from 36.6185 to 35.8103. This indicates that GhostDSC effectively suppresses unnatural artifacts and noise, producing visually more pleasant results despite the marginal sacrifice in pixel-level accuracy. The underlying reason lies in the “Ghost” mechanism: by generating redundant features through cheap linear transformations, GhostDSC imposes an implicit constraint on the feature space, preventing overfitting to pixel-wise errors while enhancing the model’s ability to generalize to real-world noise and glare patterns. Therefore, GhostDSC is indispensable for achieving an optimal balance between parameter efficiency and perceptual quality in resource-constrained scenarios.

When the LPA module is removed, the model shows notable degradation in PSNR, SSIM, and LPIPS. Specifically, PSNR drops from 21.9224 to 20.7688, SSIM drops from 0.8914 to 0.8696, and LPIPS increases from 0.4466 to 0.4891. This indicates that the absence of the attention mechanism weakens the model’s ability to focus on glare regions and edge details, making it difficult to effectively restore fine details corrupted by glare. It is worth noting that the BRISQUE metric improves after removing LPA (36.5164 vs. 43.4187), which may be due to the influence of the attention module on image texture. Nevertheless, considering other metrics, the overall contribution of the LPA module remains positive.

Overall, the full model (Ours) achieves the best results on key metrics such as SSIM, MSE, NIQE, and PIQE, fully validating the effectiveness of the GhostDSC and LPA modules in lightweight design and glare suppression. The GhostDSC module preserves feature representation capability while compressing parameters through the ghost feature generation mechanism, while the LPA module enhances the model’s perception of glare regions via parallel channel-pixel attention. Together, these modules enable the model to achieve excellent glare suppression performance while maintaining a lightweight architecture.

## 5. Conclusions

In this paper, we proposed LGSNet, a lightweight glare suppression network designed to address the critical challenges of model complexity and computational overhead in nighttime driving scenarios. To overcome the performance degradation caused by glare interference, LGSNet integrates Ghost Depthwise Separable Convolution (GhostDSC) and Lightweight Parallel Attention (LPA) to achieve an optimal balance between efficiency and restoration fidelity. Extensive experiments were conducted on the public Flare7K++ dataset and the self-constructed NRGD dataset. Quantitative results demonstrate that the proposed method, with only 7.45M parameters, outperforms or matches six representative deep learning baselines across nine full-reference and no-reference evaluation metrics, particularly excelling in perceptual quality assessment.

Despite achieving satisfactory glare removal performance and a favorable lightweight profile, this study has certain limitations. The current inference speed remains insufficient for strict real-time applications in autonomous driving systems, and the model’s robustness under extreme weather conditions requires further validation. Future work will focus on structural pruning, knowledge distillation, and TensorRT optimization to meet real-time frame rate requirements. To address these visual quality limitations, subsequent research will focus on developing specialized modules for multi-scale glare artifact suppression and designing adaptive loss functions to enhance the reconstruction fidelity of low-light regions. Crucially, the deployment performance will be verified on real edge hardware platforms in the future.

## Figures and Tables

**Table 1:** Quantitative comparison of different methods on the Flare7K++ dataset. The red, green, and blue represent the top three results.

Methods	PSNR↑	SSIM↑	LPIPS↓	MSE↓	NIQE↓	BRISQUE↓	PIQE↓
EnlightenGAN	21.3247	0.8844	0.1493	663.3174	5.4043	14.8727	29.2236
U-Net	26.5942	0.9289	0.1082	254.6440	4.8537	21.2416	37.8139
Uformer	29.6564	0.9444	0.0678	143.1108	4.8292	17.9307	34.5872
CLAHE	19.0100	0.8076	0.2088	1024.8226	4.2360	12.9004	34.6302
GAC	20.9336	0.8416	0.2244	668.1345	7.6499	44.9019	54.0619
MSRCR	21.4647	0.8878	0.1706	559.9477	5.3124	26.4145	46.1749
Ours	28.2492	0.9368	0.0933	174.8607	4.7350	20.0091	36.5721

**Table 2:** Quantitative comparison of different methods on the NRGD dataset. The red, green, and blue represent the top three results.

Methods	PSNR↑	SSIM↑	LPIPS↓	MSE↓	NIQE↓	BRISQUE↓	PIQE↓
EnlightenGAN	18.3183	0.8442	0.2664	1517.3941	18.5997	39.3279	37.6634
U-Net	23.4976	0.7441	0.1499	623.2343	7.9564	36.2114	36.2505
Uformer	24.9447	0.9131	0.1626	473.4141	18.2895	44.3654	40.5011
CLAHE	16.0054	0.7356	0.3756	1977.4971	17.7388	39.8455	49.8374
GAC	18.3760	0.6466	0.4847	1212.6741	11.1840	69.9089	54.9336
MSRCR	21.5343	0.7304	0.3104	604.7884	8.0168	36.7547	39.2956
Ours	21.9224	0.8914	0.4466	690.0233	7.1397	43.4187	35.8103

**Table 3:** Comparison of processing speed and model parameters among different methods.

Methods	Input Size	Speed↑ (fps)	#Param (M)
EnlightenGAN	512 × 512	3.45	8.636
U-Net	512 × 512	1.42	34.51
Uformer	512 × 512	1.55	20.47
CLAHE	512 × 512	20.49	–
GAC	512 × 512	25.62	–
MSRCR	512 × 512	1.15	–
Ours	512 × 512	2.20	7.45

**Table 4:** Ablation study results on the NRGD dataset.

Methods	PSNR↑	SSIM↑	LPIPS↓	MSE↓	NIQE↓	BRISQUE↓	PIQE↓
w/o GhostDSC	22.1337	0.8876	0.4456	728.3824	7.8616	44.4089	36.6185
w/o LPA	20.7688	0.8696	0.4891	701.1720	7.9969	36.5164	35.9397
**Ours**	21.9224	0.8914	0.4466	690.0233	7.1397	43.4187	35.8103

## Data Availability

The public Flare7K++ dataset is available at https://github.com/ykdai/Flare7K (accessed on 5 June 2026). The NRGD dataset and the source code for LGSNet presented in this paper are available at https://github.com/RuoyuYoung/NRGS-Net (accessed on 5 June 2026). Other public datasets used in this study, such as Flare7K++ and BDD100K, are cited accordingly.
